# Model-based plant phenomics on morphological traits using morphometric descriptors

**DOI:** 10.1270/jsbbs.21078

**Published:** 2022-02-17

**Authors:** Koji Noshita, Hidekazu Murata, Shiryu Kirie

**Affiliations:** 1 Department of Biology, Kyushu University, Fukuoka, Fukuoka 819-0395, Japan; 2 Plant Frontier Research Center, Kyushu University, Fukuoka, Fukuoka 819-0395, Japan; 3 metaPhorest (Bioaesthetics Platform), Department of Electrical Engineering and Bioscience, Waseda University, TWIns, Tokyo 162-8480, Japan

**Keywords:** plant phenotyping, morphometrics, theoretical morphology, topological data analysis

## Abstract

The morphological traits of plants contribute to many important functional features such as radiation interception, lodging tolerance, gas exchange efficiency, spatial competition between individuals and/or species, and disease resistance. Although the importance of plant phenotyping techniques is increasing with advances in molecular breeding strategies, there are barriers to its advancement, including the gap between measured data and phenotypic values, low quantitativity, and low throughput caused by the lack of models for representing morphological traits. In this review, we introduce morphological descriptors that can be used for phenotyping plant morphological traits. Geometric morphometric approaches pave the way to a general-purpose method applicable to single units. Hierarchical structures composed of an indefinite number of multiple elements, which is often observed in plants, can be quantified in terms of their multi-scale topological characteristics using topological data analysis. Theoretical morphological models capture specific anatomical structures, if recognized. These morphological descriptors provide us with the advantages of model-based plant phenotyping, including robust quantification of limited datasets. Moreover, we discuss the future possibilities that a system of model-based measurement and model refinement would solve the lack of morphological models and the difficulties in scaling out the phenotyping processes.

## Introduction

The importance of plant phenotyping techniques is increasing with the accumulation of genomic data and the widespread of sensor technologies. Recent advances have been made in plant breeding strategies, such as marker-assisted recurrent breeding and genomic selection, in response to the growing global demand for food production (Heslot *et al.* 2015, Lorenz *et al.* 2011). Although their advantages are mainly derived from genomic data, molecular breeding strategies also require phenotypic data, that is, phenotypes are not used for selection but to train a prediction model in genomic selection (Araus and Cairns 2014, Cobb *et al.* 2013, Lorenz *et al.* 2011). Plant phenotyping techniques coupled with sequencing technologies will further accelerate crop improvement strategies. In particular, an insight into the phenomics of plant morphological properties is important because many functional features depend on them, e.g., radiation interception, lodging tolerance, gas exchange efficiency, spatial competition between individuals and/or species, and disease resistance. However, there are several challenges.

**Fig. 1.**
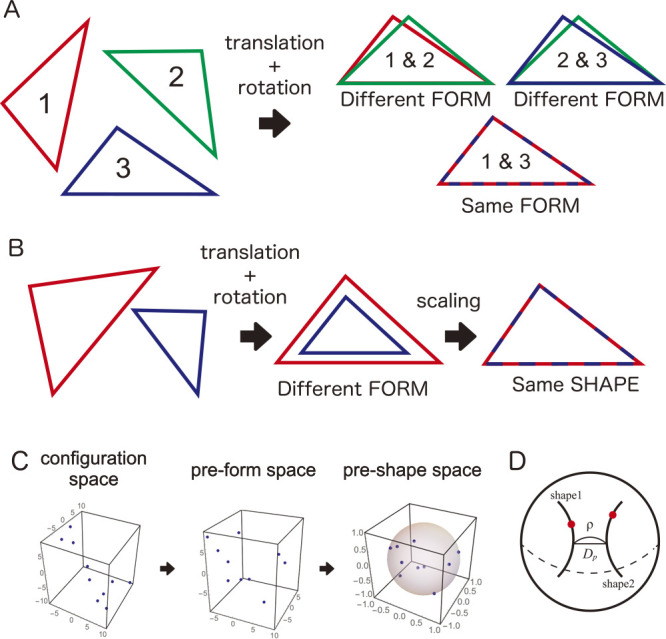
Shape as a geometric invariant. A. In geometric morphometrics, form is defined as a geometric invariant to translation and rotation. A pair of triangles 1–3 show the same form, but the pairs of 1–2 and 2–3 do not. B. The shape is defined as a geometric invariant to translation, rotation, and scaling. Two triangles cannot be matched with translation and rotation but can be matched with scaling included. C. Procrustes analysis is a process that extracts shape, i.e., removes positional, orientational, and size information. The landmark data, which are initially distributed in the configuration space, are constrained into subspaces via the Procrustes analysis. D. In the pre-shape space, the shape is represented as a trajectory of equivalence class against rotation. The differences between the trajectories are given by the great circular distance called the Procrustes distance. This figure was created based on Noshita (2021a) (Licensed under CC-BY 4.0).

**Fig. 2.**
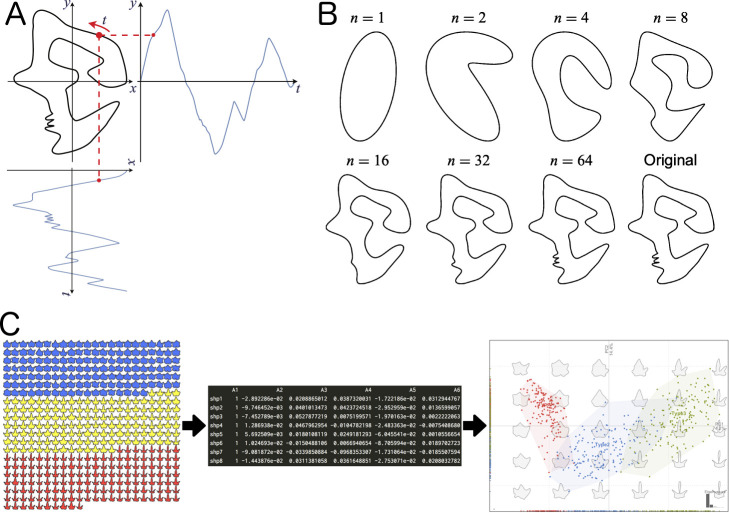
Elliptic Fourier analysis. A. An outline shape that can be quantified by the polar Fourier descriptor (left) and an outline shape that cannot be quantified by a polar Fourier descriptor (right). If the line segment between the pole and a point on the outline intersects another part of the outline, the radial distance along the polar angle is a multivalent function. B. Elliptic Fourier descriptor. The *x*- and *y*-coordinates for the parameters along the outline are modeled as other functions, respectively. C. Reconstruction of the outline shape by inverse Fourier transform. The outline shape can be reconstructed from the data, i.e., Fourier coefficients, quantified by the elliptic Fourier descriptor. The resolution of the outline shape differs depending on which order *n* of the Fourier coefficients is used. In particular, the outline is approximated by an ellipse when *n* = 1. This figure was created based on Noshita (2021b) (Licensed under CC-BY 4.0).

The first problem is the gap between the measured data acquired by various instruments and the biologically meaningful phenotypic values caused by the lack of models (e.g., quantitative representations, mathematical equations). Unlike genomic data, which are modeled and represented as strings of nucleobases (A, T, G, and C), it is not clear to what extent phenotypic data should be collected to define the phenome (Fiorani and Schurr 2013, Mahner and Kary 1997). In most cases, a simple collection of measured morphological traits, such as plant height, number of leaves, stem diameter and height, and leaf weight, and their derived statistics are insufficient to directly represent the plant morphology as a hierarchical structure combining multiple elements. Although morphological information can be easily obtained as digitized data such as images, voxels, polygons, and point clouds using several sensor technologies, such digitized data are not themselves morphological descriptors; of course, morphological parameters are included, but they require extraction via appropriate quantification methods. In geometric morphometrics, shape, an aspect of morphological traits, is defined as a geometric invariant to translation, rotation, and scaling. Based on this definition, we are able to extract the shape information of a target the morphological descriptors, which is a quantitative representation of morphological characteristics especially for further analysis, through landmark-based and outline-based models (Figs. 1, 2; see the section **Geometric morphometrics for shape of single units** for details). This requires mathematical tools for modeling, designing descriptors, and phenome data analysis of plant morphological traits (Bucksch *et al.* 2017, Furbank and Tester 2011).

The second challenge, related to the first, is the low quantitativity of phenotypic data and the resulting low throughput (Araus and Cairns 2014, Yang *et al.* 2020). It is difficult to accumulate data and replace the workforce with computational resources. Quantifying morphological properties requires tacit knowledge, and is labor-intensive and highly dependent on experts, e.g., the selection of good cultivars by breeders and tissue diagnosis by pathologists, thus making it difficult to automate and scale out such processes (Furbank and Tester 2011). This contrasts with genomics, where next-generation sequencing and bioinformatics technologies have enabled rapid and inexpensive research and development (Werner 2010). Therefore, a highly efficient phenotyping system for morphological properties is required. Some of these aspects, such as the quantitative assessment by experts and extraction of morphological features from the digitized morphology of targets, can be addressed by computer vision techniques using deep learning models (Singh *et al.* 2018). However, they may prove unreliable because the available phenotypic data to train the deep learning models are often limited, particularly at the initial stages of the breeding program and research phases. It is worthwhile to model what is to be quantified explicitly because it enables rational estimation of phenotypic values, even when the available data are limited.

In this review, we introduce three types of morphological descriptors that can be used for phenotyping of plant morphological traits: i) general-purpose geometric morphometric models, ii) topological data analysis (TDA) that can quantify structures composed of an indefinite number of elements (common in plants) for which homology is difficult to define, and iii) theoretical morphological models that capture specific anatomical structures. In reality, plants show diverse morphological characteristics on multiple scales because of their evolutional, functional, and developmental backgrounds. It is important to choose a relevant quantification model; this may extend to the development of novel models specifically for this purpose. Such model-based plant phenotyping approaches have several advantages, including robustness of quantification. Finally, we will discuss the future possibilities of an ecosystem of model-based measurement and continuous model refinement.

## Geometric morphometrics for shape quantification of single units

Geometric morphometrics can be used to extract aspects of morphological properties such as geometric invariants called form and shape (Dryden and Mardia 2016, Kendall *et al.* 2008, Zelditch *et al.* 2012). Form, which is also known as shape-and-size, is defined as a geometric invariant against translation and rotation (Fig. 1A). The shape is defined as a geometric invariant against translation, rotation, and scaling (Fig. 1B). Before the geometric morphometrics, the morphological properties of targets had been quantified using simple one-dimensional measurements, such as number, angle, length, area, volume, and ratio, combined with multivariate analysis. Beyond the series of ad-hoc measurements, geometric morphometrics achieved a direct quantification of form and shape by modeling form and shape as geometric invariants in several manners. In most studies using geometric morphometrics, the shape of single units, which are distinguished from others (e.g., single leaf, single petal, and single seed), is a primary target, and there are two well-established major approaches, known as landmark-based and outline-based morphometrics, respectively.

In landmark-based morphometrics, the morphological properties of targets are modeled as a set of landmarks, which correspond to points among specimens; the shape is extracted via a procedure removing position, size, and orientation, called the Procrustes analysis (Fig. 1C; see Dryden and Mardia (2016) and Kendall *et al.* (2008) for details). Briefly, a vector **X** represents a configuration of *k* landmarks in *m* dimensions. **X**, called the configuration vector or simply the configuration, is a *k* × *m* vector and includes the position, orientation, and size. To remove positional information, all landmark coordinate values are normalized to their centroid. Then, the size is normalized by the norm of **X**, called the centroid size. The data are then distributed on the pre-shape space, which is a high-dimensional hypersphere. The shape corresponds to a single orbit on this hypersphere as an equivalence class to the rotation, i.e., data on the orbit have the same shape but different orientations. The difference between the shapes is measured by the great-circular distance, the Procrustes distance, between these orbits (Fig. 2D).

Landmark-based morphometrics has been adapted to multiple scales from the inner-cell structure to the whole plant, including grass phytoliths (Hošková *et al.* 2021), leaves (Silva *et al.* 2012, Viscosi *et al.* 2009), lips of orchid flowers (Shipunov and Bateman 2005), whole flowers (Gardère *et al.* 2019, Savriama *et al.* 2012), and whole plants (Manacorda and Asurmendi 2018). One of the advantages of landmark-based morphometrics is its applicability to targets whose homology can be defined using shared landmarks among them, even if they consist of multiple elements. This is why the approach is applicable to whole flowers and plants. van der Niet *et al.* (2010) used landmark-based morphometrics on three-dimensional (3D) data of *Satyrium* (a genus of Orchidaceae) flowers scanned using microCT and showed an association between floral shapes and pollinator classes. Quantitative representation of shape enables further biological analyses. In a study on grapevine leaves that included landmark-based modeling and statistical analysis of biological metadata (species, developmental stages, and leaf positions), Chitwood *et al.* (2016) isolated latent shapes, which are components associated with other contexts (e.g., species and developmental stages) independent of each other, thereby indicating that developmental stages are predictive independent of species identity and vice versa. Landmark configuration is usually done manually by experts or trained people (Bookstein 1992, Dryden and Mardia 2016), but research on semi-automatic landmarking based on a small number of landmarked datasets has appeared (Pereira *et al.* 2019, Vandaele *et al.* 2018).

In outline-based morphometrics, the outlines, which are boundary lines or boundary surfaces separating the target from others, are modeled as closed functions. Elliptic Fourier analysis (EFA) is widely applied for two-dimensional modeling (Kuhl and Giardina 1982). In EFA, an outline of a target is modeled as the functions of coordinate values (*x*, *y*) on it (Fig. 2A). These functions are approximated as a Fourier series of degree *n* and quantitatively described using a set of Fourier coefficients (*a_i_*, *b_i_*, *c_i_*, and *d_i_*):

$$\begin{array}{lcl} x\left(t\right) & = & \frac{a_{0}}{2}+\sum_{i=1}^{n}\left(a_{i}\cos i\omega \pi +b_{i}\sin i\omega \pi \right),\\ y\left(t\right) & = & \frac{c_{0}}{2}+\sum_{i=1}^{n}\left(c_{i}\cos i\omega \pi +d_{i}\sin i\omega \pi \right), \end{array}$$

where *t* is a parameter indicating the position on the outline, $\omega =\frac{2\pi }{T}$ with outline perimeter *T*. More details of the outline shapes are captured with a greater degree *n* (Fig. 2B). In most cases, these coefficients are normalized (e.g., by the major axis of the ellipse defined with the first harmonic; Rohlf and Archie 1984) because the size and orientational information remain. The normalized coefficients are descriptors of the outline shapes and are often summarized using dimension reduction methods, such as principal component analysis (PCA) (Fig. 2C).

EFA has been used to quantify the shape of seeds (Ohsawa *et al.* 1998, Sakamoto *et al.* 2019, Williams *et al.* 2013), leaves (Chitwood *et al.* 2012, 2013, Iwata *et al.* 2002, 2015), petals (Yoshioka *et al.* 2004), roots (Iwata *et al.* 1998, Rellán-Álvarez *et al.* 2015), and fruits (Costa *et al.* 2009, Currie *et al.* 2000). In particular, Iwata *et al.* (2015, 2010) demonstrated the advantages of outline-based morphometrics combined with genomic data. The grain shapes of rice (*Oryza sativa* L.) were quantified using elliptic Fourier descriptors, and the descriptors or their principal component (PC) scores were used as quantitative traits. Iwata *et al.* (2010) conducted genome-wide association analysis (GWAS) of PC scores with genome-wide markers and found five significant markers associated with PC scores, including three previously reported markers. In a subsequent study, Iwata *et al.* (2015) developed non-linear regression models for genomic prediction of grain shape based on genome-wide single nucleotide polymorphism (SNP) genotypes.

Spherical harmonic analysis is available for the modeling of 3D morphological structures (Ritchie and Kemp 1999, Shen *et al.* 2009, Styner *et al.* 2006). In this approach, coordinate values (*x*, *y*, *z*) on an outline (closed surface) of a target are approximated as a linear combination of spherical harmonics:

$$\begin{array}{lcl} x\left(\theta ,\phi \right) & = & \sum_{l=0}^{\infty }\sum_{m=-l}^{l}\left(c_{x,l}^{m}Y_{l}^{m}\left(\theta ,\phi \right)\right),\\ y\left(\theta ,\phi \right) & = & \sum_{l=0}^{\infty }\sum_{m=-l}^{l}\left(c_{y,l}^{m}Y_{l}^{m}\left(\theta ,\phi \right)\right), \end{array}$$

and

$$z\left(\theta ,\phi \right)=\sum_{l=0}^{\infty }\sum_{m=-l}^{l}\left(c_{z,l}^{m}Y_{l}^{m}\left(\theta ,\phi \right)\right),$$

where $Y_{l}^{m}\left(\theta ,\phi \right)$ is a spherical harmonic function of degree *l* and order *m*, and *θ* and *ϕ* are parameters indicating a position on the spherical coordinates, respectively. An outline shape is quantitatively described using a set of the expansion coefficients $\left(c_{x,l}^{m},c_{y,l}^{m},c_{z,l}^{m}\right)$ , similar to EFA. Spherical harmonic analysis has been used to describe the 3D outline shapes of citrus fruits (Ding *et al.* 2000), agricultural grains (beans, chickpeas, and maize) (Radvilaitė *et al.* 2016).

The extraction of outlines from digitized data (e.g., images, volume data) can be achieved via a relatively simple image analysis, which is user-friendly because it does not require an explicit definition of homology, such as a configuration of landmarks. Several useful geometric morphometrics tools have been provided as R packages, e.g., for landmark-based morphometrics (Adams and Otárola-Castillo 2013, Dryden 2021), combined landmark-based and outline-based morphometrics (Bonhomme *et al.* 2013), desktop applications for outline-based morphometrics (Iwata and Ukai 2002) and landmark-based morphometrics, including phylogenetic analysis (Klingenberg 2011), and web applications (Dujardin and Dujardin 2019).

## TDA will overcome the issue of ill-defined homology

Recently, topological data analysis (TDA), which refers to statistical methods for extracting structures from data using topological concepts, has been widely available for the quantitative representation of morphological properties in several fields. These include material science (Hiraoka *et al.* 2016, Saadatfar *et al.* 2017), biochemistry (Kovacev-Nikolic *et al.* 2016, Xia and Wei 2014), developmental biology (McGuirl *et al.* 2020), and neuroscience (Bendich *et al.* 2016, Saggar *et al.* 2018). In particular, persistent homology (PH) analysis captures topological features in data on a multi-scale and enables the quantification of abstract topological features.

**Fig. 3.**
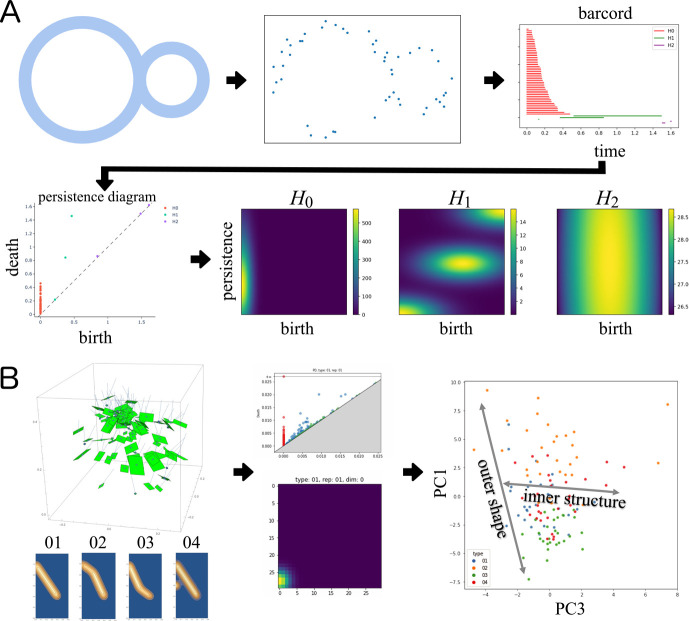
Persistent Homology Analysis. A. An example of persistent homology (PH) analysis of point cloud data generated based on a structure. The birth-death profiles of homology classes are summarized in a persistence diagram (PD). Several vectorized representations are derived from the PD for further statistical analysis; persistence images (PIs) are shown here. B. An example of PH analysis of simulated plant foliage. The foliage structures were represented as point cloud data corresponding to leaf positions. Based on the point cloud data, the PD was generated based on the Čech complex. Using PIs, the differences among foliage structures were recognized in the data space generated by principal component analysis.

PH analysis calculates the topological characteristics by constructing a filtration of simplicial complexes based on input data, e.g., images, point clouds, and graphs. A simplex is a generalization of arbitrary dimensions of points, line segments, triangles, and tetrahedrons; that is, the smallest convex set composed of linearly independent *k* vectors given by *k* + 1 points in *n*-dimensional Euclidean space **R***^n^* is called a *k*-simplex. Furthermore, *l*-simplexes, defined as the convex sets of *l* + 1 vertices taken from the vertices of *k*-simplex are called faces of *k*-simplex, which is a generalization of faces of polyhedrons. A set of simplexes *K* is called a simplicial complex if a face *α* of a simplex β∈K belongs to *K* and γ∩δ is a face of both *γ* and *δ* if not γ∩δ=∅ for *γ*, δ∈K . In PH analysis, the homology classes, which are structures with holes, are calculated in several dimensions at the simplicial complex using an increasing sequence of the simplicial complex called a filtration, constructed based on the data. Persistence diagrams (PDs) depict the birth-death profiles of the homology classes during filtration. Based on the PD, the dissimilarity between two data points can be represented topologically (Edelsbrunner and Harer 2009). For practical use, several vectorized representations are derived from the PD, such as the persistence landscape (Bubenik 2015), persistence image (PI) (Adams *et al.* 2017), and Betti curve (Umeda 2017). Fig. 3A shows an example of a workflow for quantitatively describing the topological features using PH analysis; the PD and the PIs were calculated from point cloud data sampled from a structure.

Recently, PH analysis has been applied to quantify plant morphological traits. For example, Li *et al.* (2018a) successfully captured multi-scale morphological features of diverse and disparate leaf shapes among 141 families of seed plants, which include different numbers of lobes and leaflets, using PH analysis, and represented them in the common morphospace; it is difficult to define their homology using only the landmark-based and/or outline-based morphometrics approaches mentioned in the previous section. Li *et al.* (2017) discussed the possibility of PH analysis for branching patterns (plant shoots, roots, and clusters) and the versatility of a morphometrics tool for plant structures. Li *et al.* (2018b) demonstrated that the topological features of leaf shapes, leaf serrations, and tomato roots, obtained via PH analysis, improved the associations between genotype and phenotype, that is, clear and unique quantitative trait loci (QTLs) were detected by utilizing topological features as opposed to conventional traits (and their multivariate derivatives). Fig. 3B shows an example of the quantification of four foliage structures using the PD and the PIs. For each probability distribution of leaf occurrence on the radial plane corresponding to the different structures, 30 point cloud data representing the leaf positions were simulated (Fig. 3B left). The point cloud data were quantified as the PDs and the PIs (Fig. 3B middle). The PCA was conducted for the PIs, and different distribution patterns were recognized among the four structures in the data space (Fig. 3B right).

Although there are no established routines for analyzing plant structures consisting of an indefinite number of elements, TDA may be a breakthrough technology for quantifying such structures in plant science (Amézquita *et al.* 2020, Bucksch *et al.* 2017). Several open-source libraries and packages are available (e.g., Tauzin *et al.* 2021, The GUDHI Project 2021).

## Theoretical morphological modeling of specific anatomical structures

If we recognize mathematical, geometric, developmental, or other morphological rules, developing a model for describing specific anatomical structures could be an effective approach. Theoretical morphological approaches make it possible to organize morphometric information in a model-based manner and indirectly evaluate features that are difficult to measure directly. It is possible to generate hypothetical morphologies based on the models and evaluate their morphological features and functions to understand better why they do or do not exist; that is, to identify the potential constraints on morphological diversity. These strengths were already recognized in the first papers on theoretical morphological approaches, and Raup’s work on theoretical morphological models of shells (Raup 1966, Raup and Michelson 1965) discussed morphological diversity and its functional and structural constraints. Previous work on land plant evolution (Niklas 1994, 1999) and leaf shapes (Runions *et al.* 2017) have also adopted theoretical morphological approaches. However, there is a limited number of theoretical morphological studies compared to morphometric approaches because researchers attempting to conduct a theoretical morphological study are required to find and/or develop a model for mimicking the target morphological traits. Here, we introduce several theoretical morphological studies on phyllotaxis, branching patterns, and flowers as examples.

Several theoretical studies on phyllotaxis adopt the theoretical morphological approaches. Douady and Couder (1996a, 1996b, 1996c) conducted experiments and numerical analyses with theoretical models that work on principles of spiral phyllotaxis proposed by Hofmeister (1868). These studies showed several diagrams that visualized the relationship between the model parameters that regulate development and the spiral phyllotactic patterns produced by those parameters. Although they might not have intended to draw morphospaces, some basic ideas are shared; that is, these diagrams can also be used to visualize the potential clusters and the constraints of the pattern. One of the latest studies developed from the Douady-Couder model demonstrated that phyllotactic patterns were mapped on a parametric diagram and showed how to transit from one pattern to another, which cannot be achieved in the Douady-Couder model (Yonekura *et al.* 2019). In terms of its applications related to floral morphologies, the number of perianths and the symmetry properties have been respectively investigated using the Douady-Couder model and the phase diagram to visualize the pattern transition (Kitazawa and Fujimoto 2015, Nakagawa *et al.* 2020).

Branching patterns is another typical subject of theoretical morphological studies, in particular their geometric regularity and growing rules. L-system, a cellular automaton accepting both cell division and hierarchical structures, mimics branching patterns and their growth by using generated strings and a growth grammar (Lindenmayer 1968a, 1968b, 1971). For example, consider a finite string consisting of symbols, including an empty string, which models a cellular array with specific states; a grammar is a set of rewriting rules for symbols and operates each symbol in the string. From an initial string called the axiom, the string is rewritten recursively based on the grammar and new strings are generated in each step. The L-system describes a hierarchical branching structure by representing certain symbols as biological components (e.g., cells, organs, and other anatomical structures) and geometric indicators (e.g., bifurcation and changing direction) via turtle interpretation. Early studies (Lindenmayer 1968b, 1971) successfully mimicked the branching pattern of *Callithamnion roseum*, a red alga, and the leaf development of *Tortula acaulon*, a moss, using the context-free L-system (0 L-system). In subsequent studies, extended models of the L-system have been developed, e.g., stochastic L-system (Eichhorst and Savitch 1980), parametric L-system (Chien and Jürgensen 1992), open L-system (Měch and Prusinkiewicz 1996), and relational growth grammar (Kniemeyer *et al.* 2007). A wide range of plant structures, such as trees, herbaceous plants, flowers, leaves, and their developmental processes, have also been generated using these models (Prusinkiewicz and Lindenmayer 1990). The L-system and its extended models have been used in combination with plant/crop models, which are models for mimicking the dynamics of growth stages and physiological processes, as functional structural plant models; this allows feedback among structures, physiological processes, and surrounding environments (Barczi *et al.* 2008, Kniemeyer *et al.* 2007, Pradal *et al.* 2008, Vos *et al.* 2010).

One of the difficulties in quantifying plant morphology is the hierarchical composition of several types of elements in an indefinite number, which type of morphology is often observed in the flower shape. Although this characteristic, i.e., a flower consists of multiple elements, such as petals, makes it difficult to define the homology between objects and apply geometric morphometrics, there are cases in which the morphology can be summarized and described as model parameters. As noted previously, there are several known mathematical models of floral structure based on phyllotaxis models (Kitazawa and Fujimoto 2015, Nakagawa *et al.* 2020). We expect some general-purpose theoretical morphological models to be developed by combining them with existing morphogenesis simulations, even though theoretical morphological models have been used in the analysis of floral morphology in only a few cases. Here, we introduce an example of a theoretical morphological model of a flower. Although this model does not parameterize flower development and only describes the whole flower morphology, it represents a typical application of the theoretical morphological approach.

**Fig. 4.**
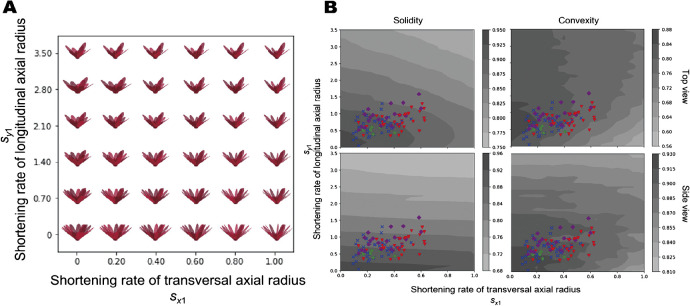
Morphospace and feature spaces mapped on the morphospace of a theoretical morphological model of *Nymphaea* flower. A. Morphospace of the theoretical morphological model. Each parameter regulates the gradual change of transverse or longitudinal length of the tepal. B. Feature spaces mapped on the morphospace. The convexity, which is the ratio of perimeter lengths of the convex hull over that of a silhouette of the theoretical morphological model, and the solidity, which is the ratio of areas of the silhouette of the model over that of the convex hull, are displayed. The upper two spaces were calculated for the top-viewed silhouette of the model and the lower spaces were calculated for the side-viewed silhouette. Each feature index showed a different pattern for the change of parameters, therefore, it suggests the “trade-off” involved in designing floral morphology via selective breeding. This figure was created based on partially modified Figs. 3 and 5 in Kirie *et al.* (2020) (Licensed under CC-BY 4.0).

Conventionally, floral morphologies are given qualitative descriptions by breeders or specialists using natural language or combinations of simple measurements in floriculture. Thus, it is difficult to interpret the meaning and actual appearance quantitatively. Kirie *et al.* (2020) proposed a theoretical morphological model for *Nymphaea* (water lilies) flowers to describe the morphological diversity of horticultural cultivars; the floral morphology was reduced to three hierarchical rules, namely (1) morphologies of tepals, (2) the spiral phyllotaxis, and (3) the blooming state. To demonstrate the versatility of this model, a theoretical morphospace was generated by varying several parameters, especially those related to the gradual change in size and shape through a series of tepals. The flower shapes of the measured cultivars were mapped into some subspaces of the theoretical morphospace, that is, one can estimate the occupation pattern of real floral morphologies in a subspace spanned by measurable parameters (Fig. 4). Consequently, it was suggested that typical flower shapes could be classified. The study included a survey of the parameters or features of parts as well as features of the whole morphology. Both the convexity and solidity of whole flowers were calculated as global features for each shape sampled from the theoretical morphospace using silhouettes of theoretical floral morphologies and their convex hulls. The results suggest that some flower shapes can also be classified based on global features. Moreover, these features showed different sensitivities in response to changes in the model parameters and the direction of projection. It seems conceivable that such a theoretical approach could translate qualitative descriptions made by floricultural breeders to quantitative or machine-readable ways and facilitate the design of new morphologies.

## Model-based phenotyping improves the robustness of estimation in limited datasets

The model-based phenotyping approaches described in the previous sections help us to extract morphological traits and improve the robustness of the estimation when datasets are limited or small. If we know the constraint and diversity of the morphological traits of targets, it will serve as a geometric prior to restricting the morphospace to be explored.

**Fig. 5.**
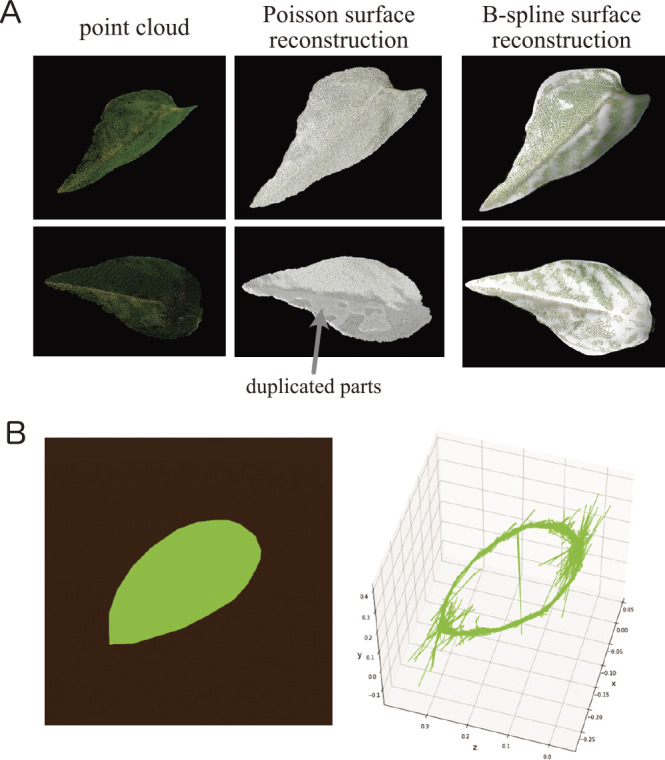
Three-dimensional (3D) recontraction of leaf morphology based on explicit assumptions. A. Point cloud data of the leaf surface (left) and results of surface reconstruction using Poisson reconstruction (middle) and B-spline surface fitting (left). In this case, the duplicated reconstruction was recognized as the result of Poisson reconstruction, which is often used for this purpose, and resulted in over-estimation of the leaf area. The B-spline surface fitting avoided the problem by assuming that a leaf is a single two-dimensional surface in a 3D space. B. Simulation data of a leaf (left) and a leaf reconstructed using curve features (right). The contours and reconstructions of the simulation data are almost identical. There is a lot of noise at both ends of the slope. The contour is represented as a set of curve fragments.

**Fig. 6.**
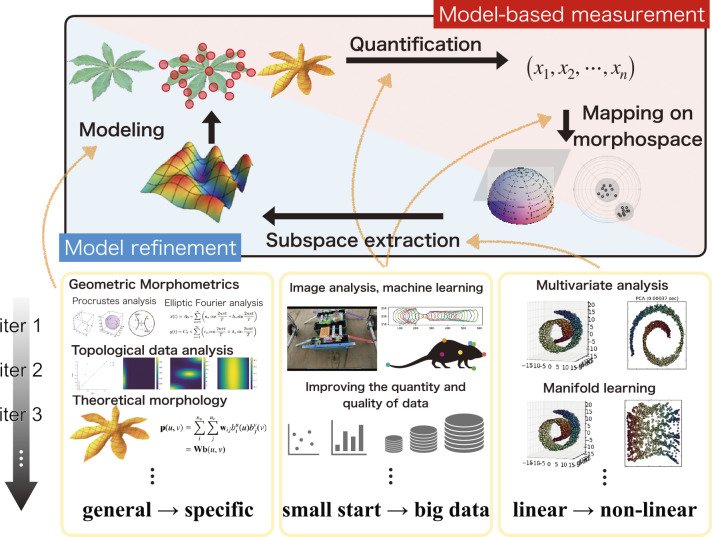
A concept for a continuous improvement cycle for plant phenotyping consisting of model-based measurements and model refinements. Morphological data were obtained using the morphological descriptors. A subspace of the morphospace in which the data are distributed is extracted via dimension-reduction methods. A new morphological model is proposed to cover the subspace. Using the new model would enable the next measurement. Thus, the dataset can be expanded continuously, presenting more options for data analysis, including non-linear methods. More precise and robust plant phenotyping procedures could be achieved by iterating the continuous improvement cycle.

Assuming a two-dimensional leaf structure, model-based phenotyping allows us to make more robust measurements of leaf traits for surface reconstruction. For example, consider the reconstruction of a leaf surface using point cloud data and estimations of the morphological traits; point cloud data digitized from real plants contain noise that is difficult to remove completely, even using denoising filters. Therefore, the surface was reconstructed using Poisson surface reconstruction (Kazhdan *et al.* 2006), resulting in an overestimation of the leaf area (Fig. 5A). A possible solution is to assume *a priori* that the leaf is a single (bounded) surface. For example, Fig. 6A shows a leaf surface reconstructed via B-spline surface fitting with B-spline curve trimming (Mörwald 2013), i.e., the leaf surface was model as a piece of surface; the leaf surface reconstruction was clearly improved, and the approach provided robust measurements despite the point cloud data containing some noise. Several methods have been proposed to provide robust measurements of leaf morphological traits, e.g., by modeling the leaf surface as triangular meshes (Dornbusch *et al.* 2007), a set of small patches (Pound *et al.* 2014), or as another piecewise polynomial function (Kempthorne *et al.* 2015).

Another interesting example is that of leaf contour shape extraction in a 3D space. Although a leaf is a structure that exists in a 3D space, the leaf shape is usually quantified and evaluated using two-dimensional measurements. Capturing such a one-dimensional closed curve in a 3D space as an outline shape is challenging because of the difficulty of accurately estimating the boundary based on point clouds reconstructed using other approaches, such as structure from motion (SfM) and multi-view stereo (MVS). For example, in a study that presented a method for visualizing leaf veins and contours from laser-scanned point cloud data, Sun *et al.* (2011) extracted the structure outline from the mesh, but the mesh did not extend to the boundary points. Curve-based 3D reconstruction is a candidate for modeling the outline as a one-dimensional closed curve in a 3D space directly (Fabbri and Kimia 2010). Unlike SfM and other point-based methods, curve-based reconstruction uses the curves in the image as features for 3D reconstruction. Therefore, it can reconstruct thin structures, such as leaves and branches, as more stable curves instead of as points (Li *et al.* 2018c). Moreover, because it reconstructs the outlines directly, it is possible to obtain clear leaf boundaries. To improve the accuracy of curve-based reconstruction, it is recommended to perform deep learning-based instance segmentation, e.g., Mask R-CNN (He *et al.* 2017), on leaf outlines instead of conventional edge detection methods such as a differential filter that picks up noise such as leaf veins. Fig. 5B shows an example of curve-based 3D reconstruction on simulated data, that is, images and masks of the leaves acquired by 48 cameras. The outline was extracted as a set of curved fragments reconstructed from pairs of mask images. Although the apical portion of the leaf was unstable in comparison to the original 3D mesh, these curved fragments could be merged using a curve averaging method for further improvements (Usumezbas *et al.* 2016). Thus, it is expected that structures that are difficult to observe in 3D space directly could be handled by direct modeling, especially the thin structures of plants themselves.

## Conclusion and future perspective

In this review, we introduced several morphological descriptors, summarized the major concepts of mathematical models and the theories behind them, and presented various examples of their applications. Using geometric morphometric approaches, the morphological properties of single units in plants were quantified as shape (or form) without over- or underestimation (Figs. 1, 2). Even if plants show a hierarchical structure composed of an indefinite number of multiple elements, TDA will enable the representation of such complex morphological features from the perspective of topological characteristics (Fig. 3). Theoretical morphological models also enable the quantification of complex plant morphology in cases where we discover mathematical, geometric, developmental, or other morphological rules (Fig. 4). Although finding rules in plant morphology is generally the result of trial and error, data accumulation will accelerate the process (Cao 2017). On the other hand, model-based phenotyping also accelerates data accumulation because of its robustness when applied to limited datasets (Fig. 5). These model-based phenotyping approaches coupled with sequencing technologies are promising future for crop improvement because the complex morphological properties, which are difficult with conventional qualitative and subjective evaluations, can be analyzed as quantitative traits and predicted based on genomic data (e.g., Hiraoka *et al.* 2016, Iwata *et al.* 2015).

However, the morphological traits of plants show complex and hierarchical structures and such traits are not always covered by the models introduced in this review. As mentioned in the section **Theoretical morphological modeling of specific anatomical structure**, there are not many available theoretical morphological models, and further development of a model for mimicking the target morphological traits often required. In the research phase, when improving hypotheses continuously using limited data and a poorly systemized quantification workflow, it is important to extract biologically meaningful information from each experiment and provide feedback to the next as much as possible (e.g., Campbell *et al.* 2018, Rahaman *et al.* 2015). Such a problem would be partially solved by continuously developing morphological models using model-based phenotyping approaches (Fig. 6). To improve the model continuously, we measure morphological traits based on the model, extract a subspace of the morphospace by analyzing the data obtained from the measurement, and develop and propose a new model that covers the subspace. Initially, we can start with generic methods, such as geometric morphometrics, to collect data with limited prior knowledge of targets. We then extract a subspace of the morphospace by analyzing the obtained data using linear statistical methods such as PCA. A new morphological model that covers the extracted subspace of the morphospace is proposed, and we then proceed to the next measurement based on the new morphological model. Even if the measurement fails partially or contains a large amount of noise, robust quantification will be possible using the constraints of the model as prior knowledge. Thus, the dataset can be expanded continuously. As the data increase, more options become available for data analysis, such as nonlinear dimension reduction (e.g., Cayton 2005). This implies the possibility of extracting a subspace that is more appropriate given the diversity of morphological data. This may lead to the proposal of a theoretical model that can cover the obtained subspace. ﻿The integration of all the constituent elements into a continuous improvement cycle is required for phenotyping of plant morphological properties. This includes edge devices for model-based measurements, a web application programming interface providing the backend of morphometric processes and data accumulation, and user interfaces to conduct mathematical and statistical analyses.

## Author Contribution Statement

K.N., H.M., and S.K. wrote the first draft of the manuscript. K.N. performed the demonstration of geometric morphometrics, TDA, and leaf surface reconstruction. H.M. performed the virtual plant model generation and curve-based reconstruction. S.K performed the theoretical morphological analysis of flower shapes. K.N. supervised the study. All authors have read the manuscript and provided comments to improve the manuscript.
